# Identification of Novel EXT Mutations in Patients with Hereditary Multiple Exostoses Using Whole‐Exome Sequencing

**DOI:** 10.1111/os.12660

**Published:** 2020-04-15

**Authors:** Chao Liang, Yong‐jie Wang, Yu‐xuan Wei, Yang Dong, Zhi‐chang Zhang

**Affiliations:** ^1^ Department of Orthopaedics Shanghai Jiao Tong University Affiliated Sixth People's Hospital Shanghai China; ^2^ Department of Orthopaedics, National Cancer Center/National Clinical Research Center for Cancer/Cancer Hospital & Shenzhen Hospital Chinese Academy of Sciences and Peking Union Medical College Shenzhen China

**Keywords:** Bone tumor, EXT1, EXT2, Hereditary multiple exostoses, Whole‐exome sequencing

## Abstract

**Objective:**

To find novel potential gene mutations other than EXT1 and EXT2 mutations, to expand the mutational spectrum of EXT and to explore the correlation between clinical outcome and genotype in patients with hereditary multiple exostoses (HME).

**Methods:**

The study recruited seven families diagnosed with multiple osteochondromas (MO). Family histories and clinical information were collected in detail through comprehensive physical and image examination. Patients with deformities and functional limitations were classified as “severe” and the remaining without functional limitations were classified as “mild,” in accordance with previous study. Whole‐exome sequencing (WES) was performed on a total of 13 affected individuals, 1 available unaffected relative, and 10 healthy unrelated individuals. Sanger sequencing was used to validate the screened mutations. Finally, the structural change in protein caused by pathogenic mutations was analyzed using information from the relevant database online and we attempted to correlate clinical phenotype with genotype in patients with HME.

**Results:**

Other than EXT1 and EXT2, no novel potential gene mutations were found through WES. We identified nine heterozygous mutations in EXT1 or EXT2. Of these mutations, four have not been reported previously. These are c.996delT in exon 2 of EXT1 (family 1), c.544C > T in exon 3 of EXT2 (family 2), c.1171C > T in exon 7 of EXT2 (family 5), and c.823_–_824delAA in exon 5 of EXT1 (family 7). The other five mutations have already been reported in previous works. It was surprising that we found two mutation sites, in exon 2 and exon 5, respectively, of EXT1 in 1 patient diagnosed with MO, when his father had two mutation sites, in exon 6 and exon 5, respectively, of EXT1 and EXT2 (family 4). In addition, 1 patient showed degeneration, while his father only exhibited slight symptoms (family 7). In our study, among 51 affected patients in seven families, the sex ratio (male *vs* female) was 58.9% (*n* = 30) *vs* 41.2% (*n* = 21). Male patients seemed to show more severe symptoms compared to females, but because the sample was small, we did not obtain statistically significance results.

**Conclusion:**

Whole‐exome sequencing to screen pathogenic gene mutations was applied successfully. Although no third‐gene mutation associated with HME was found, a total of nine mutations across EXT1 and EXT2 were identified, four of which are novel. Our results expand the mutational spectrum of EXT and can be used in genetic counseling and prenatal diagnosis for patients with MO.

## Introduction

Hereditary multiple exostoses (HME) is a rare autosomal inherited skeletal disease. The prevalence is estimated to be 1:50 000 among North Americans^1^. The main symptom of HME is the presence of exostoses, with patients tending to be diagnosed before the age of 12 years. Multiple exostoses usually develop within the perichondrium of young vertebrates, mainly at the ends of long bones, although occasionally in the ribs and hips. On average, an affected patient is burdened with six exostoses^2^. For most patients, the large size and specific location of exostoses frequently cause short stature, bone deformity, pain, and joint limitation. A consensus has not yet been reached in clinical classification. Marina *et al*. designed a simple and efficient clinical classification (I–III) to evaluate the severity of the phenotype on 289 HME patients, which has been validated using the Switching Neural Network. The assessment system depended on the deformities, the functional limitations, and the number of affected sites^3^.

Malignant transformations are relatively rare. If exostoses continue to grow after the growth plate has closed and the cartilage cap is more than 2 cm, there is a high risk of malignant change in approximately 5% of patients^4^. Medical treatment for the disease still does not exist, although several researchers have found a few particular molecules involved in related signal pathways. Local surgical excision is the only effective treatment but still cannot resolve the deformity and help patients to overcome the psychological obstacle. Third generation *in vitro* fertilization technology is promising. The technology allows experts to select healthy embryos to block the inheritance through next generation screening^5^.

Gene sequencing on excision samples from affected individuals led to the discovery of the EXT1 gene, located on Chromosome 8 and the EXT2 gene, located on Chromosome 11. Both encode Golgi‐resident glycosyltransferases responsible for the synthesis of heparan sulfate (HS)^6^, ^7^. The low level of HS has a vital role in the initial development of exostoses. Heterozygous mutations in EXT1 and EXT2 cause a deficiency of synthesis HS of approximately 50%, but formation of exostoses still requires a “second hit” to lower the HS levels so that affected cells eventually become tumorigenic^8^. In the end, a small number of mutated cells recruited wild cells and they together developed into an osteochondroma^9^. However, it is uncertain how the deficiency of HS influences downstream regulatory signals. The steep decrease in HS causes excessive bone morphogenetic protein (BMP) signaling that provokes ectopic chondrogenesis and dampens fibroblast growth factor (FGF) signaling, which has the opposite effect on the formation of osteochondroma^10^. At least 85% of cases are associated with heterozygous mutations in either EXT1 or EXT2^11^. In the remaining 15% of cases, no genomic alterations were detected in either EXT1 or EXT2. This indicates that other genomic alterations, such as EXT3, EXTL1, EXTL2 and EXTL3, may also play a role^12^, ^13^. However, their existence remains disputed as the technology rapidly develops.

Due to the limitations that have affected gene sequencing technology up to now, researchers used specific gene probes covering all exons in either EXT1 or EXT2 to detect the EXT mutations^14^, ^15^. As a result, other potential gene mutations may have been overlooked. With the rapid development of whole‐exome sequencing (WES), more genes that may be associated with genetic diseases have been screened^16^.

Whole‐exome sequencing has considerable power to identify novel causal genetic variants because it can cover all of the exon regions. All exons consist of approximately 22 000 genes responsible for encoding protein, accounting for 1.5% of the DNA. Compared to whole genome sequencing (WGS), WES is more cost effective so that the technology can be applied in prenatal diagnosis. It can identify the disease phenotypes in advance if the mutated gene shows up in the public database. At the same time, it provides a more precise diagnosis, improving our ability to counsel families^17^.

With regards to the relationship between clinical outcome and genotype in patients with HME, Francannet *et al*. suggested that the most severe groups of the disease and malignant degeneration were associated with EXT1 mutations. For sex‐dependent differences, the ratio appears to be higher in males, and male patients usually present with severe symptoms, possibly due to the sex hormone differences^2^. In a domestic study, Li *et al*. showed that patients with EXT1 mutations are prone to showing more severe symptoms than those with EXT2 mutations in terms of stature, deformity, and limitation of joints, whereas there were no gender‐related differences in his study^18^.

In this study, we performed WES on 13 affected patients, 1 available relative, and 10 unrelated individuals to screen the potential gene mutation and expand our knowledge of the mutation hotspots of EXT. Finally, we collected clinical information to explore the correction between clinical outcome and genotype in patients with HME.

## Patients and Methods

### *Patients*

**Figure 1:**
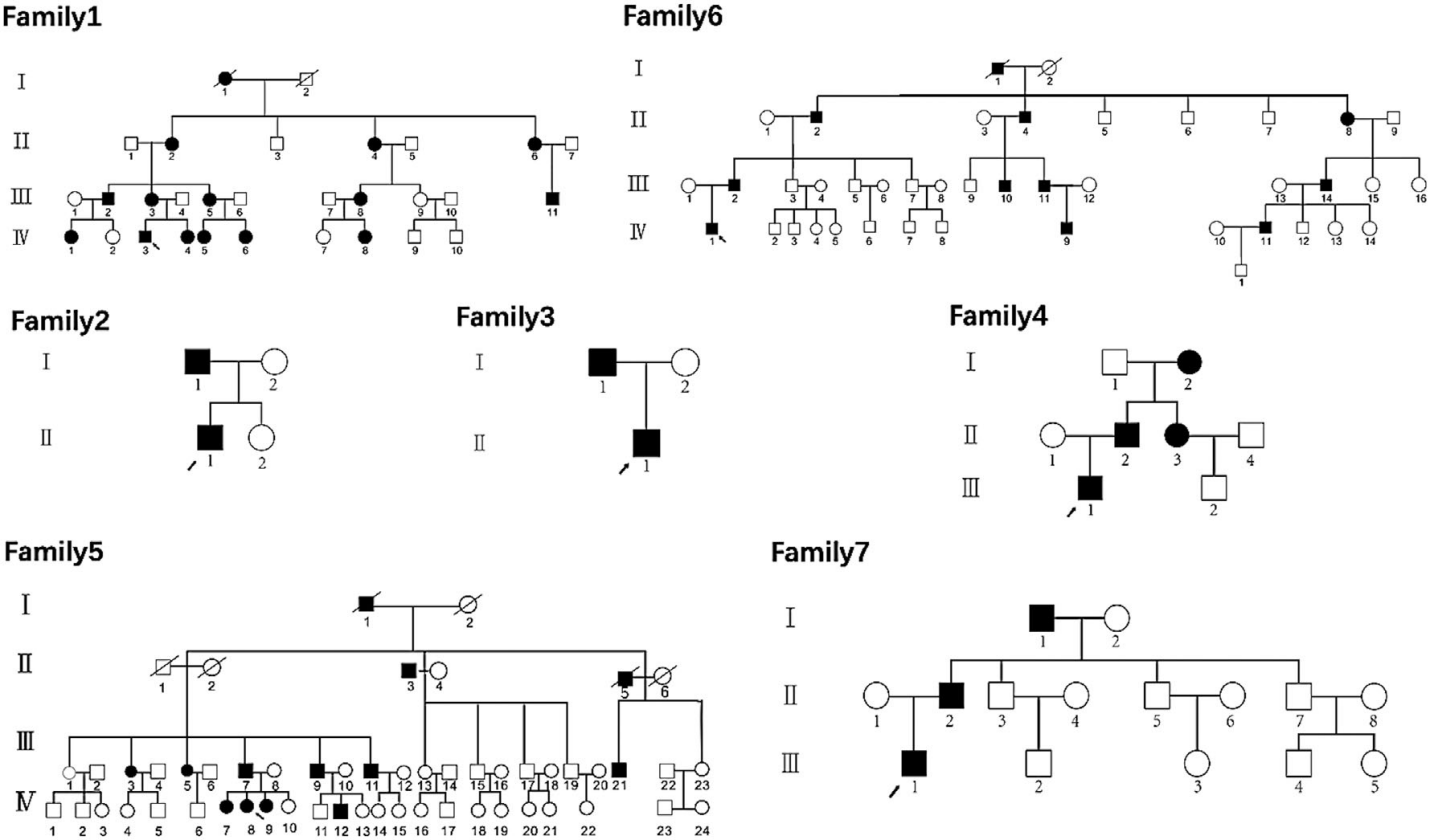
Pedigree of seven families with multiple osteochondroma (MO). Black symbols represent the affected individuals and open symbols represent unaffected individuals. Circles and squares represent females and males, respectively. Arrows identify the probands in each family.

There were 13 patients with osteochondromas (MO), 1 available relative, and 10 unrelated individuals who were evaluated with WES. All of the families belonged to the Han ethnic group. MO was diagnosed with at least two exostoses that can be detected by radiological observation. Blood samples from these families were obtained. Family histories and clinical information were collected in detail. The clinical information and pedigree are shown in Table 1 and Fig. 1, respectively. This study was approved by the ethics committee of Shanghai Jiao Tong University Affiliated Sixth People's Hospital (Shanghai, China).

**Table 1:** Clinical data and mutations identified in EXT1 and EXT2 from MO patient

Family no.	Patient no.	Gender	Age (years)	Mutation of site	cDNA change	Protein change	Type of change	Novel
F1	IV4	M	7	EXT1/Exon 2	c.996delT	p.Phe333Serfs*26	Frameshift	Yes
F1	IV3	F	11	EXT1/Exon 2	c.996delT	p.Phe333Serfs*26	Frameshift	Yes
F1	III3	F	35	EXT1/Exon 2	c.996delT	p.Phe333Serfs*26	Frameshift	Yes
F2	II1	M	9	EXT2/Exon 3	c.544C > T	p.Arg182*	Nonsense	Yes
F3	II1	M	13	EXT1/Exon10	c.2053C > T	p.Gln685*	Nonsense	No
F3	I1	M	42	EXT1/Exon10	c.2053C > T	p.Gln685*	Nonsense	No
F4	III1	M	19	EXT2/Exon 5	c.896G > A	p.Arg299His	Missense	No
				EXT2/Exon 2	c.8C > T	p.Ala3Val	Missense	No
F4	II2	M	45	EXT2/Exon 5	c.896G > A	p.Arg299His	Missense	No
				EXT1/Exon 6	c.1469delT	p.Leu490Argfs*9	Frameshift	No
F5	IV8	F	20	EXT2/Exon 7	c.1171C > T	p.Gln391*	Nonsense	Yes
F5	III7	M	53	EXT2/Exon 7	c.1171C > T	p.Gln391*	Nonsense	Yes
F5	IV9	F	24	EXT2/Exon 7	c.1171C > T	p.Gln391*	Nonsense	Yes
F6	IV1	M	20	EXT2/Exon 2	c.514C > T	p.Gln172*	Nonsense	No
F7	III1	M	20	EXT2/Exon 5	c.823_–_824delAA	p.Lys275Thrfs*10	Frameshift	Yes

F, female; m, male; MO, multiple osteochondromas

**Table 2:** Clinical information of patients

Family number	Patient number	Numbers of affected bone	Deformity	Limitation of joint	Times of therapy
F1	IV4	>10	Severe	Severe	0
F1	IV3	>10	Mild	No	0
F1	III3	<10	Severe	Severe	1
F2	II1	>10	Mild	No	0
F3	II1	>10	Severe	Severe	1
F3	I1	<10	Mild	No	0
F4	III1	>10	Severe	Severe	2
F4	II2	>10	Mild	No	0
F5	IV8	<10	Mild	No	0
F5	III7	>10	Severe	Severe	2
F5	IV9	<10	Mild	No	0
F6	IV1	>10	Severe	Severe	1
F7	III1	>10	Severe	Severe	2

### *Exome Capture and Sequencing*

Genomic DNA was extracted from peripheral blood cells according to the manufacturer's standard procedure (MagPure Buffy Coat DNA Midi KF Kit). gDNA was broken into 100–500 bp fragments using a BGI enzymes kit (Segmentase, BGI), The 280–320 bp fragments were collected using magnetic beads. We then added “A” base at 3′ terminal after repairing ending so that the fragments could pair “T” base with a special adapter. Extracted DNA was amplified using a ligation‐mediated polymerase chain reaction (LM‐PCR). After purification and enrichment, one single individual DNA library was built. A mean exome coverage of more than 98% was obtained. The sequencing depth was greater than 100× for capture regions. Finally, the qualified products were sequenced with PE100 + 100 on MGISEQ‐2000 (BGI, China).

### *Sanger Sequencing*

The primers were designed for standard polymerase chain reaction assays using Primer 3 software Version 0.4.0 (http://bioinfo.ut.ee/primer3-0.4.0/). The amplified DNA fragments were directly sequenced by Sanger sequencing. DNASTAR software was used to analyze the sequencing results through comparison with the reference sequences.

### *Analysis of Variants*

Whole‐exome sequencing reads were mapped to human genome hg19 with the software tool BWA; variants were identified using a set of bioinformatic tools called a Genome Analysis Toolkit (GATK); genetic variants were annotated using the software tool ANNOVAR. Novel EXT mutations were recognized with the Human Gene Mutation Database (http://www.hgmd.cf.ac.uk/). The structural changes in proteins caused by pathogenic mutations were analyzed using UniProtKB (http://www.uniprot.org/) and MutationTaster (http://www.mutationtaster.org/).

## Results

### *Description of Patients*

In family 1 (F1), the proband (IV‐3) was an 11‐year‐old boy. He had multiple exostoses near both knees and elbows and exhibited the obvious genu valgum deformities that are common complications of MO. His sister (IV‐4), who suffered from the same disease, exhibited less pronounced symptoms than the proband. Their mother (III‐3), who was 35 years old, underwent resection of exostoses on both distal femurs in 2015 due to restricted knee joint movement.

**Figure 2:**
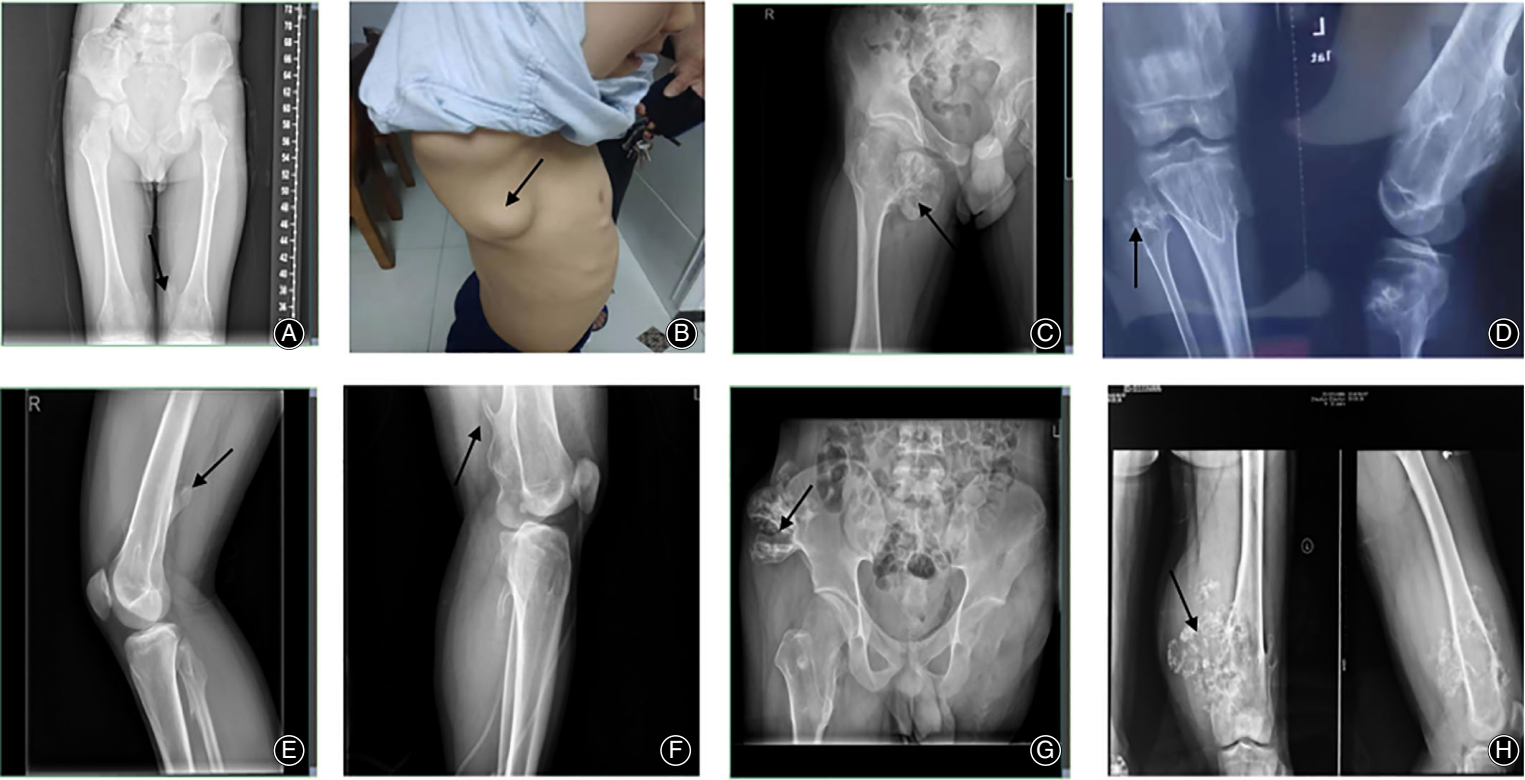
Appearance and radiology of the probands. (A) F1: X‐rays shows multiple exostoses on bilateral distal femur; (B) F2: One obvious bony prominence on his right rib; (C) F3: A huge exostosis on the proximal femur affecting the activation of hip joint; (D–F) (F4–F6, respectively): The X‐ray plain film shows multiple exostoses near the knee requiring surgery; (G) F7: a cauliflower‐like exostosis on the top of the pelvis; (H) F7: The plain radiograph of the distal femur showed extensive osteolytic lesions and obvious calcifications with a soft‐tissue mass.

In family 2 (F2), the proband (II‐1) exhibited multiple exostoses around the joints and ribs. There was one huge exostosis on the right rib, as shown in Fig. 2B. His father (I‐1) underwent surgery on his humerus in 1994, but it continued to grow until his growth plates closed.

**Figure 3:**
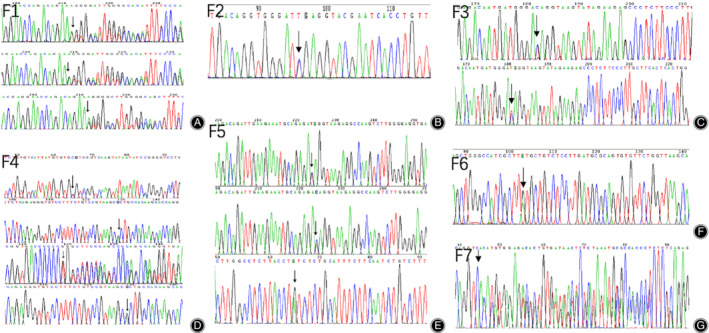
Mutation analysis. (A) a frameshift mutation, c.996delT in exon 2 of EXT1; (B) a nonsense mutation, c.544C > T in exon 3 in EXT2; (C) a nonsense mutation. c.2053C > T in exon 10 in EXT1; (D) a missense mutation, c.896G > A in exon 5 of EXT2, a point mutation, c.8C > T in exon 2 of EXT2, a deletion, c.1469delT, in exon 6 of EXT1; (E) a nonsense mutation, c.1171C > T in exon 7 of EXT2; (F) a nonsense mutation, c.514C > T in exon 2 of EXT2; (G) a frameshift mutation, c.823_–_824delAA in exon 5 in EXT2.

In family 3 (F3), the proband (II‐1) underwent surgical removal of the exostoses on the right proximal femur at the age of 13 because of their great size, which affected his hip joint. However, his father (I‐1) exhibited only slight symptoms and his life was not seriously affected by the disease.

In family 4 (F4), the proband (III‐1) with exostoses at multiple sites, including the left proximal humerus, bilateral shoulder, proximal and distal femur, and proximal tibia and fibula, had undergone resection of exostoses two times. The first surgery was performed on the left distal humerus in 2012 and the second on both proximal tibias in 2016; however, his father (II‐2) did not undergo any surgery until this present work.

In family 5 (F5), the proband (IV‐8) presented more severe symptoms than her two sisters. When she was 15 years old, she started to feel pain in her knee joint because of the huge exostoses on the right proximal femur. However, her sister (VI‐7), who also suffered from the disease, had no pain or other obvious manifestations. Her father bore severe limitation of joints and deformity.

In family 6 (F6), the proband (IV1) had experienced pain on his right distal femur for 4 years, and he decided to address it by undergoing resection. His father (III2) experienced no manifestation other than bony prominences.

In family 7 (F7), the proband (III‐1) was the only one who underwent malignant transformation among any of the 13 patients. In April 2018, a plain radiograph of the distal femur showed extensive osteolytic lesions with a soft‐tissue mass. Subsequently, the patient underwent surgery on his distal femur to remove the huge osteochondroma after biopsy, as shown in F2 H. However, 3 months later, his left thigh become more swollen and he felt more pain than he had felt ahead of the surgery.

Because the main artery and nerve had been invaded, there was no choice but to amputate the affected lower limb. A diagnosis of the secondary peripheral chondrosarcoma was confirmed by pathology. Besides this exostosis, there was another huge exostosis on his pelvis, as shown in F2 G. It was amazing that his father, who suffered from the disease, only exhibited slight symptoms. The details of clinical information were shown in table 2 and Fig. 2. We included patients with deformities and functional limitations in the severe group; the remaining were included in the mild group, in accordance with a previous paper^3^.

### *Identification of Mutations*

In this study, we reported the results of WES in seven families with MO. We found a total of nine different heterozygous mutations in EXT1 and EXT2. As anticipated, we did not find any EXT mutations in the 1 unaffected relative or in any of the 10 unrelated individuals. Here, four of these nine mutations have not been reported in any previous study (Table 1) and five of these nine mutations are recurrent (Table 1)^19^, ^20^, ^21^, ^22^. Next we performed Sanger sequencing to confirm these mutations, as shown in Fig. 3.

None of 13 patients were negative for either EXT1 or EXT2 mutations. In F1, a deletion mutation in exon 2 of EXT1, p.Phe333Serfs*26 (c.996delT), was present in the 3 people. In F2, a nonsense mutation in exon 3 of EXT2 (c.544C > T) that were detected led mutation, p.Gln685*(c.2053C> T), in exon 10 of EXT1 was found to similarly cause the premature protein truncation.

In F4, the point mutation (c.896G> A), one recurrent mutation, in EXT2 was present in father and son. Significantly, another point mutation in exon 2 of EXT2, p.Ala3Val (c.8C > T), was present in the son and a deletion in exon 6 of EXT1, p.Leu490Argfs*9(c.1469delT), was found in the father. Both of these have been reported previously^21^, ^22^. According to the inherited pattern, c.8C > T in exon 2 of EXT2 was novel for the proband (III‐1 of F4). In F5, a point mutation, c.1171C > T, of exon 7 in EXT2 caused a nonsense mutation. In addition, a p.Gln172* (c.514C > T) mutation of exon 2 in EXT2 was found in F6 and a frameshift mutation caused by c.823_‐_824delAA of exon 5 in EXT2 was identified in F7.

### *Comparison*

From 51 affected patients in seven families, the sex ratio (male *vs* female) was (58.9% [*n* = 30] *vs* 41.2% [*n* = 21]). The ratio has slight bias because a few members did not find they suffered from this disease due to their mild symptoms, especially for women. We attempted to find sex‐dependent differences in the patients who received comprehensive examinations. Male patients seemed to show more severe symptoms compared to female patients (55.6% [*n* = 5] *vs* 25% [*n* = 1], *P* = 0.559) when we classified patients with deformities and functional limitations as the severe group; the remaining were included in the mild group. Then we explored the genotype–phenotype correlation of hereditary multiple exostoses. There was no difference in phenotype between individuals with EXT1 mutations and individuals with EXT2 mutations (33.3% [*n* = 2] *vs* 50% [*n* = 4], *P* = 1) using the previous method. Because the sample size was small, we could not obtain a significant difference.

## Discussion

Although the molecular mechanisms associated with HME are not fully understood, it is clear that HME is predominantly provoked by mutation in the EXT gene. In family 4, it was surprising that we found two mutation sites, in exon 2 and exon 5, respectively, of EXT1 in the son diagnosed with MO, but his father had two mutation sites, in exon 6 and exon 5, respectively, of EXT1 and EXT2, whereas his mother is normal. Maybe the son accumulated the novel mutation and inherited the normal chromosome from his mother. The severity of the disease varies, even within the same family whose affected members all share the same mutation^23^. One previous study showed that the malignant transformation from the preexisting osteochondroma depends on the wild‐type cells with functional EXT. In other words, EXT‐independent mechanisms are involved in the occurrence of secondary peripheral chondrosarcoma^24^. This is why the proband (F7 III‐1) underwent degeneration while his father only exhibited slight symptoms.

Germline mutations of the EXT1 and EXT2 genes, which cause the disease, can be identified in approximately 85% of affected patients with MO. Single‐exon and multi‐exon region deletions of the EXT1 or EXT2 genes have been found in approximately 10% of all tested cases by multiple ligation‐dependent amplification (MLPA)^11^. For the remaining mutation‐negative and deletion‐negative cases, it is possible that the presence of mosaic deletions causes EXT mutation in some cells that cannot be detected by MLPA, or these patients may carry a mutation located in the non‐coding region in EXT1 or EXT2 that can affect EXT pre‐mRNA splicing, mRNA maturation, or transportation^25^. In this study, samples are small. In theory, we cannot rule out the existence of other gene mutations in EXT‐negative families. It is expected that a suitably modified version of existing screening technology may help identify other pathological mutations in EXT‐mutation‐negative patients. In addition, the number of individuals who joined the study in every family is limited; therefore, we cannot study other factors that can affect the development of disease even if they have the same mutation site.

In conclusion, although we did not find any third gene mutation capable of causing MO using WES, we identified four novel EXT gene mutations and confirmed WES was helpful in extending the EXT mutational spectrum. Our results can help in screening for pathological mutations and support genetic counseling and subsequent prenatal diagnosis.
